# GeriPOP: Older Adults Population Health Serious Game

**DOI:** 10.1093/geroni/igab046.2812

**Published:** 2021-12-17

**Authors:** Gunjan Manocha, Casey Morton, Jeremy Holloway, Scott Brewster, Joseph Wood, Richard Van Eck, Donald Jurivich

**Affiliations:** 1 University of North Dakota, Grand Forks, North Dakota, United States; 2 Triad Interactive Media, Brooklyn, New York, United States

## Abstract

Health professionals have limited opportunities to learn about population health in their curriculum. With a shortage of geriatricians nationwide, health care systems need different ways to provide evidence-based geriatric care. To address both these shortcomings, a serious game, called GeriPOP has been developed to allow trainees to explore the impact of assessment and management of principles of geriatric care (the 4Ms+) on quality of life, health, longevity, and health care costs by applying them to a virtual older adult population. Trainees assume the role of a system manager who is asked to explore ways to optimize health outcomes and lower costs. They develop their population health plan around a framework of Geriatric 4Ms+ and apply it in a virtual panel of older adult patients that move longitudinally into different age bands (65-74; 75-84; 85+). As the game progresses, a dashboard helps trainees track the impact of their treatment decisions across the population. Several levels of play allow trainees to explore various issues intersecting with aging such as gender, diversity, social determinants, and multiple chronic conditions. Periodic debriefings and explanatory pop ups during the game allow trainees to further explore evidence–based Geriatrics. The game engages health care trainees to strengthen their knowledge of Geriatrics through exploration of systems change. Future study is needed on whether Geri POP changes learner attitudes, future clinical practice or healthcare outcomes.

